# Supplementary material for: Synthesis and Properties of the Gallium-Containing Ruddlesden-Popper Oxides with High-Entropy B-Site Arrangement
Source: Materials (Basel). 2022 Sep 19;15(18):6500. doi: 10.3390/ma15186500 (PMC9503551; doi:10.3390/ma15186500)
Supplement: Supplementary file 1 [file materials-15-06500-s001.zip › materials-1917256-supplementary.pdf]

materials MDPI

Supplementary Materials

Synthesis and Properties of the Gallium-Containing Ruddlesden-Popper Oxides with High-Entropy B-Site Arrangement

Juliusz Dąbrowa [1,*], Jan Adamczyk [1], Anna Stępień [2,3], Marek Zajusz [1], Karolina Bar [1], Katarzyna Berent [4] and Konrad Świerczek [2,3]

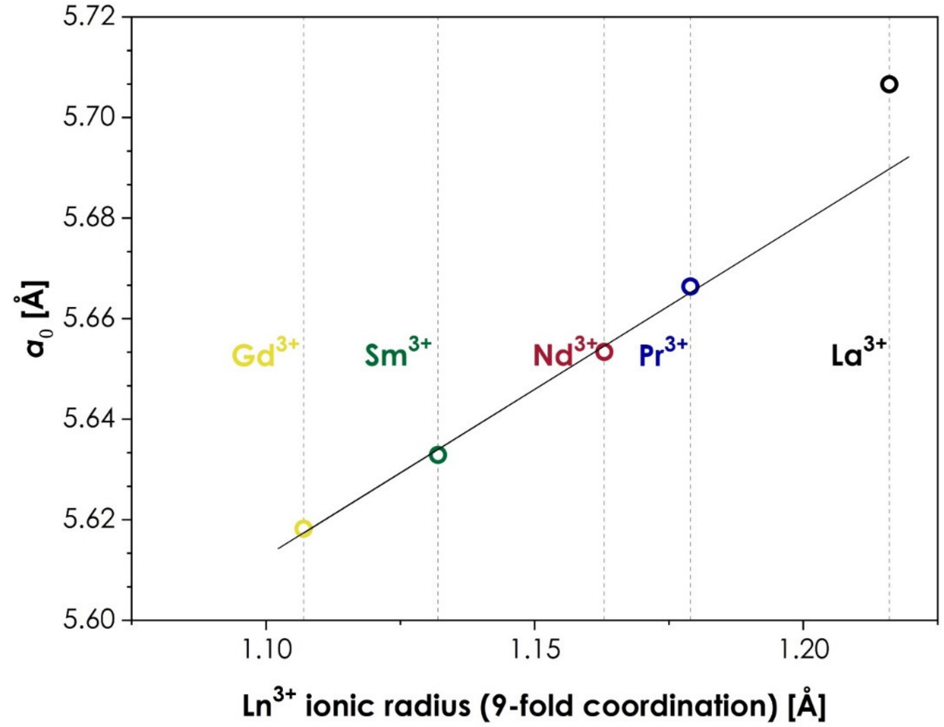

Figure S1. The dependence between the normalized, quasi-cubic lattice parameter a_0 and the ionic radii of the Ln^{3+} ions for the $LnSr(Co,Fe,Ga,Mn,Ni)O_4$ (Ln = La, Pr, Nd, Sm, or Gd) series. The linear regression line based on the Gd-, Sm-, Nd-, and Pr-based compositions is extrapolated on the value of ionic radius corresponding to La^{3+}.

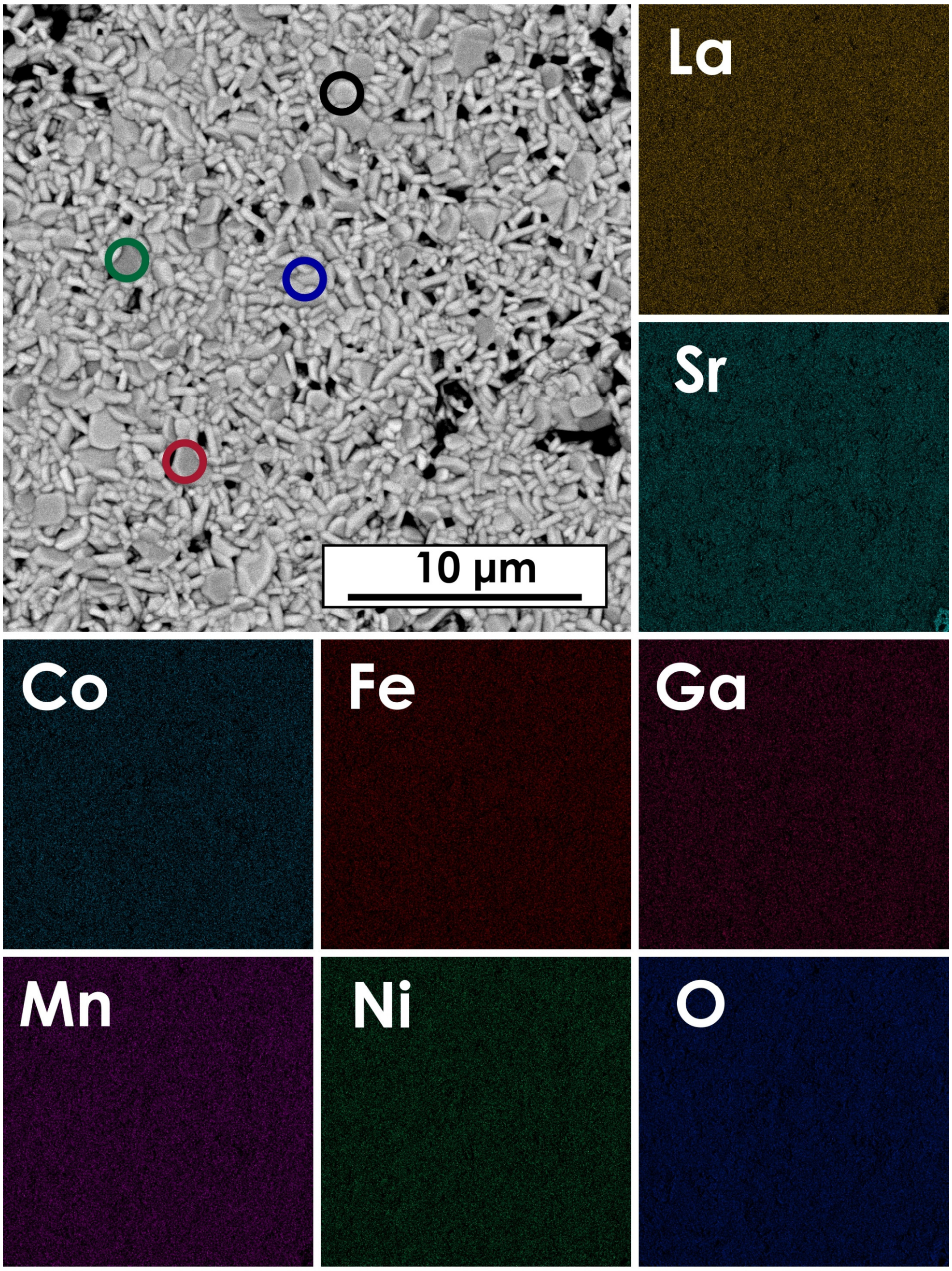

	La	Sr	Co	Fe	Ga	Mn	Ni	O
○ (black)	16.3	15.4	2.4	2.1	2.5	2.2	2.5	56.7
○ (red)	20.2	17.4	2.1	2.5	4.1	2.3	2.8	48.7
○ (green)	18.5	16.6	1.8	1.8	2.1	2.3	2.2	54.7
○ (blue)	19.1	16.1	2.6	2.7	3.6	2.6	2.9	50.6

Figure S2. The results of EDS mapping and point analysis for the LaSr(Co,Fe,Ga,Mn,Ni)O_4 pellet sintered at 1200 °C for 20 h.

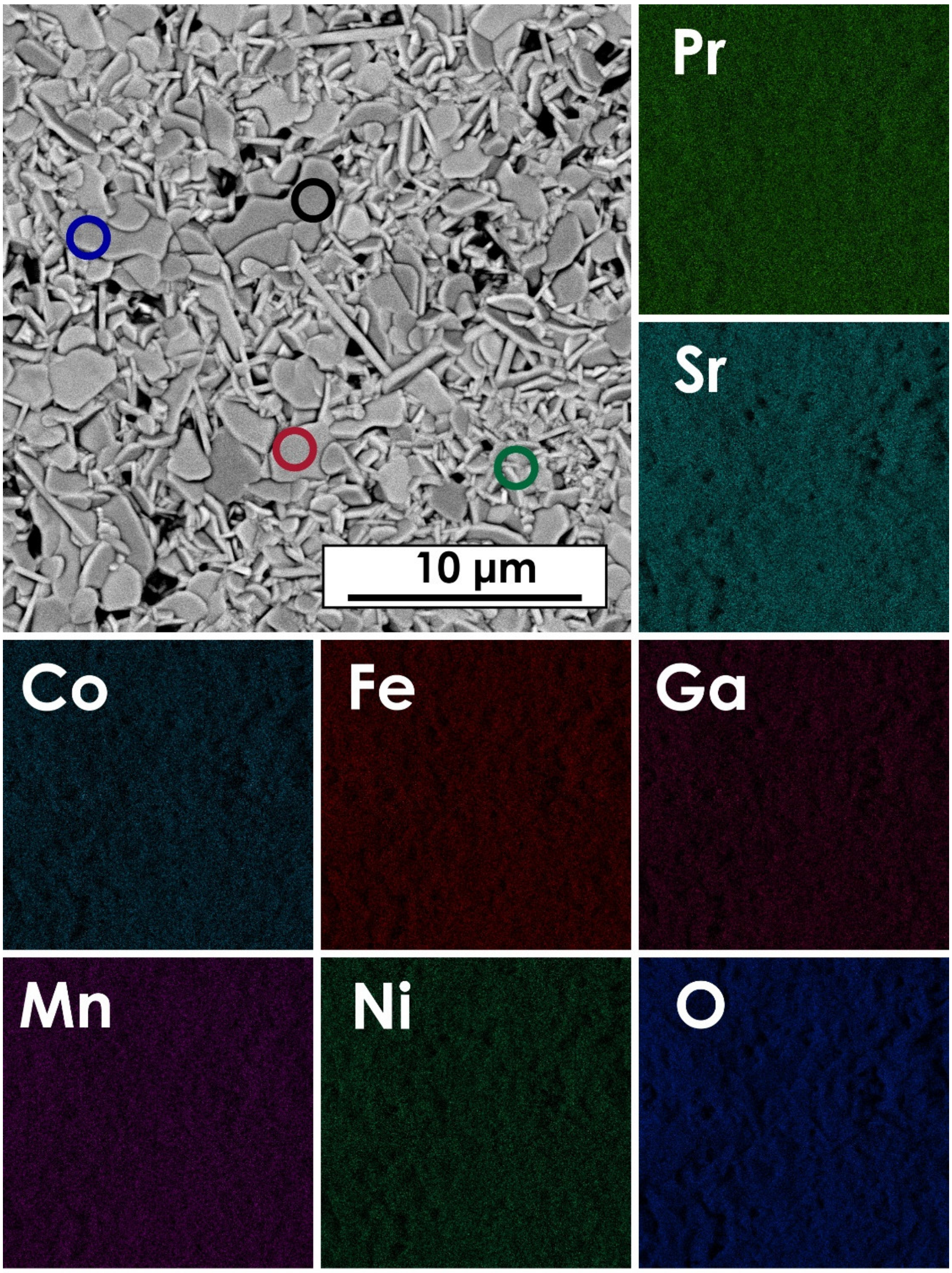

	Pr	Sr	Co	Fe	Ga	Mn	Ni	O
○ (black)	20.6	17.6	2.0	2.3	2.9	2.3	2.2	50.2
○ (red)	20.6	17.5	2.8	3.0	3.0	2.2	2.6	48.4
○ (green)	16.5	15.7	2.1	2.0	2.2	1.6	2.0	58.1
○ (blue)	17.8	16.5	1.8	2.1	3.9	1.7	2.2	54.1

Figure S3. The results of EDS mapping and point analysis for the $PrSr(Co,Fe,Ga,Mn,Ni)O_4$ pellet sintered at 1200 °C for 20 h.

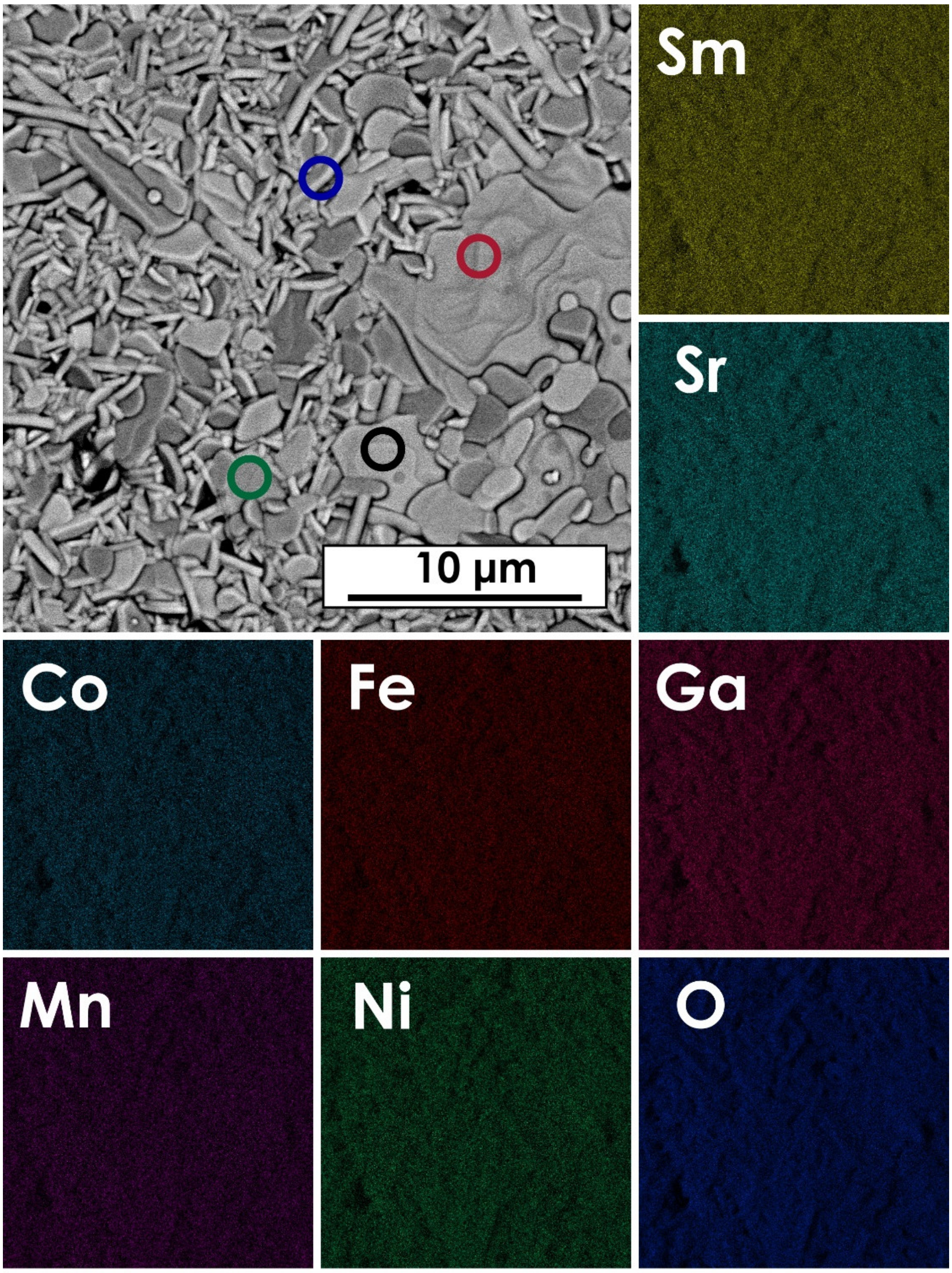

	Pr	Sr	Co	Fe	Ga	Mn	Ni	O
○	19.3	16.6	1.8	2.2	3.4	2.2	2.1	52.4
○	18.8	16.3	2.1	2.5	2.2	2.1	2.1	53.9
○	18.5	16.2	2.5	2.4	3.0	2.7	2.7	52.1
○	16.3	15.4	1.9	1.7	2.1	1.7	1.6	59.4

Figure S4. The results of EDS mapping and point analysis for the SmSr(Co,Fe,Ga,Mn,Ni)O_4 pellet sintered at 1200 °C for 20 h.

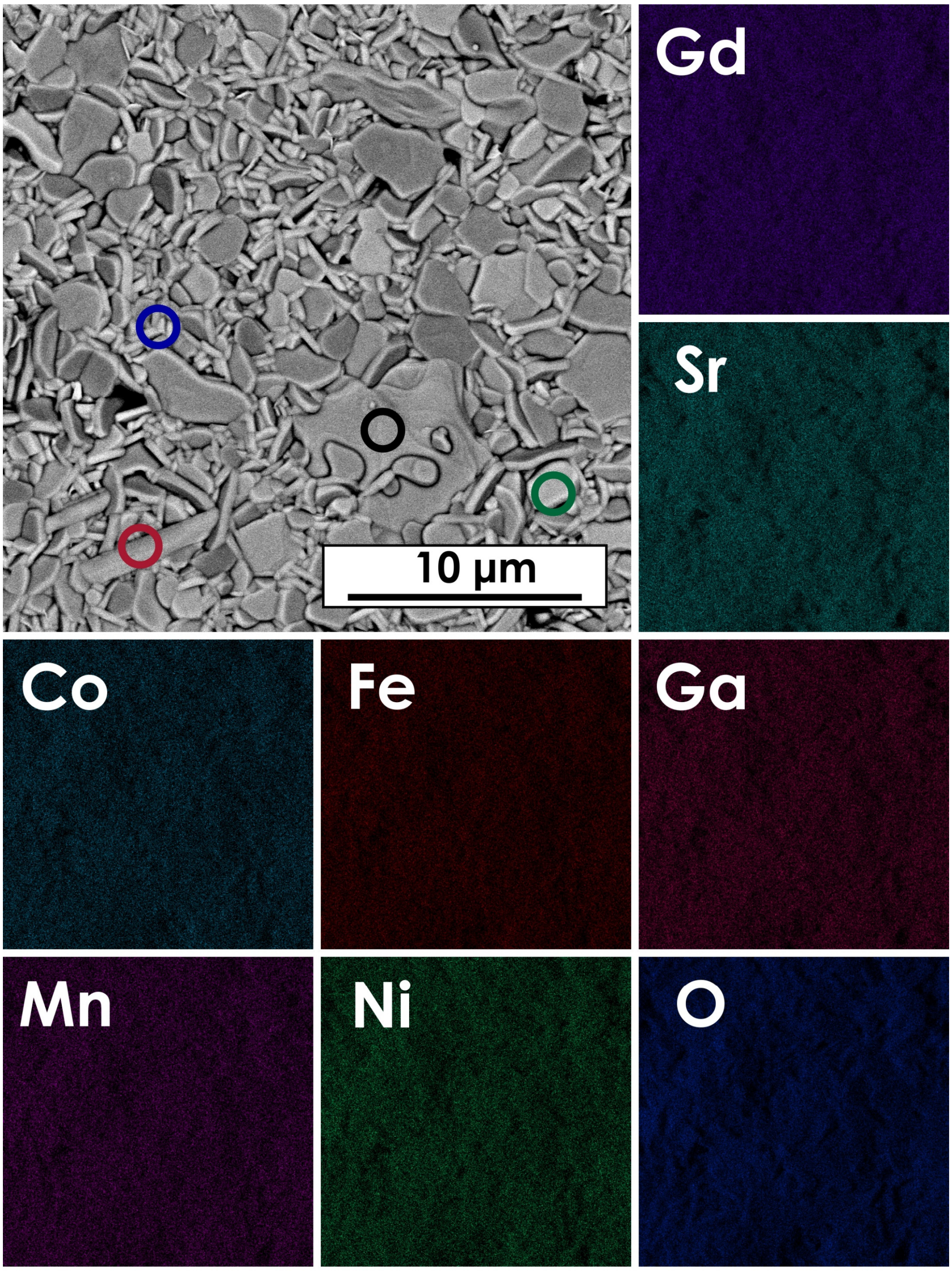

	Gd	Sr	Co	Fe	Ga	Mn	Ni	O
○	20.2	16.4	2.6	2.3	1.7	2.5	2.8	51.5
○	19.1	15.7	2.0	2.2	2.4	2.1	2.3	54.3
○	18.3	15.6	2.3	2.3	2.4	2.3	1.8	55.0
○	19.4	15.5	2.1	2.1	2.6	2.0	2.5	53.8

Figure S5. The results of EDS mapping and point analysis for the GdSr(Co,Fe,Ga,Mn,Ni)O_4 pellet sintered at 1200 °C for 20 h.

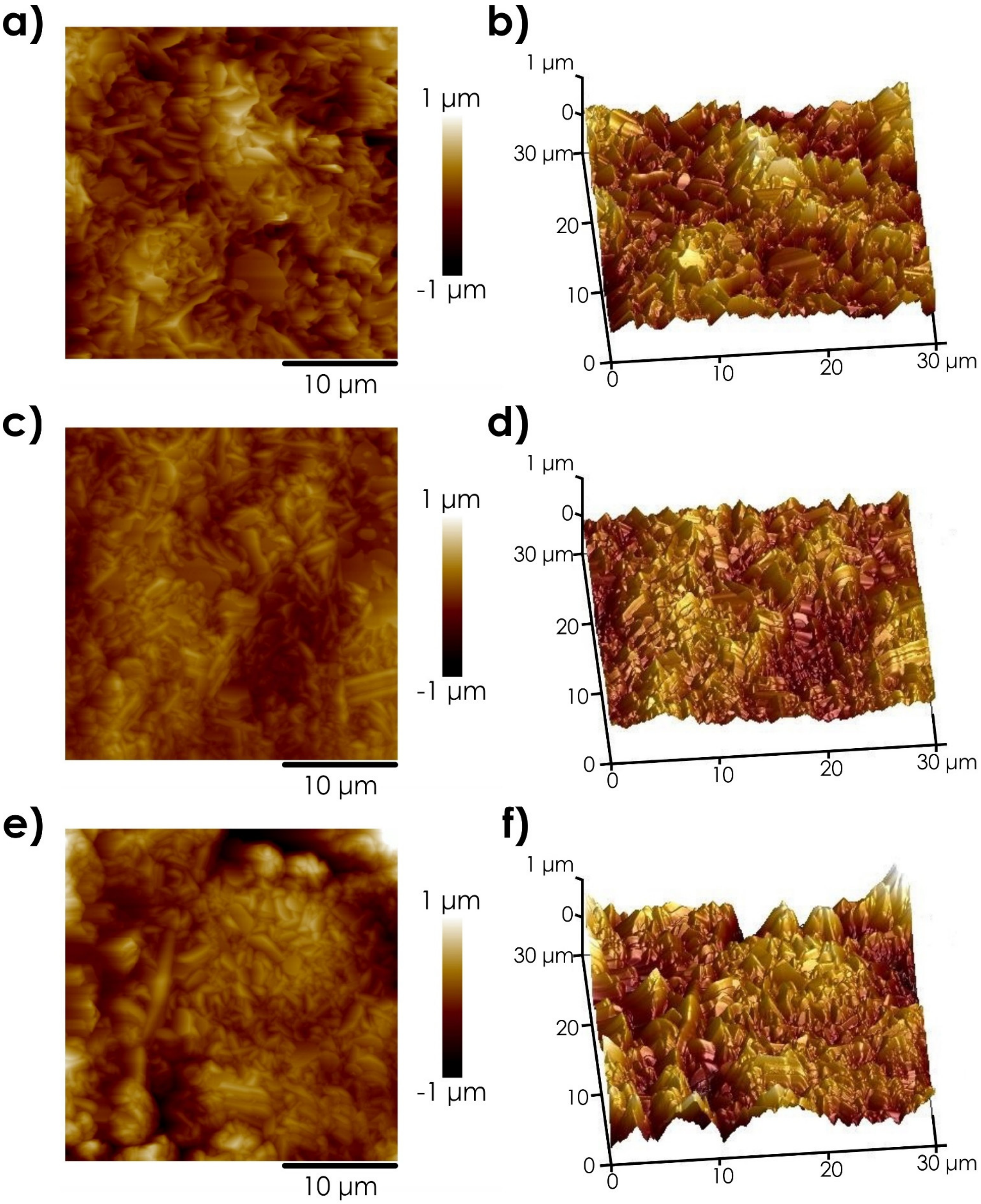

Figure S6. The results of the AFM mapping (tapping mode): (**a**) 2D micrograph of the PrSr(Co,Fe,Ga,Mn,Ni)O_4 pellet; (**b**) 3D micrograph of the PrSr(Co,Fe,Ga,Mn,Ni)O_4 pellet; (**c**) 2D micrograph of the NdSr(Co,Fe,Ga,Mn,Ni)O_4 pellet; (**d**) 3D micrograph of the NdSr(Co,Fe,Ga,Mn,Ni)O_4 pellet; (**e**) 2D micrograph of the SmSr(Co,Fe,Ga,Mn,Ni)O_4 pellet; (**f**) 3D micrograph of the SmSr(Co,Fe,Ga,Mn,Ni)O_4 pellet.

Table S1. The results of the iodometric titration measurements conducted for the LnSr(Co,Fe,Ga,Mn,Ni)$O_{4+\delta}$ (Ln = La, Pr, Nd, Sm, or Gd) series.

Composition	Powder mass[g]	Volume of titrant [ml]	Oxygen content	Oxygen non-stoichiometry	Average oxygen non-stoichiometry	Deviation
LaSr(Co,Fe,Ga,Mn,Ni)$O_{4+\delta}$	0.0508	12.017	4.006	0.006	0.006	0.004
	0.0513	12.017	4.002	0.002		
	0.0513	12.217	4.009	0.009		
PrSr(Co,Fe,Ga,Mn,Ni)$O_{4+\delta}$	0.0507	12.017	4.009	0.009	0.015	0.006
	0.0508	12.217	4.015	0.015		
	0.0509	12.417	4.021	0.021		
NdSr(Co,Fe,Ga,Mn,Ni)$O_{4+\delta}$	0.0507	11.817	4.006	0.006	0.008	0.005
	0.0506	12.017	4.014	0.014		
	0.0509	11.817	4.004	0.004		
SmSr(Co,Fe,Ga,Mn,Ni)$O_{4+\delta}$	0.0510	11.817	4.011	0.011	0.013	0.003
	0.0508	11.817	4.012	0.012		
	0.0502	11.817	4.017	0.017		
GdSr(Co,Fe,Ga,Mn,Ni)$O_{4+\delta}$	0.0501	11.617	4.019	0.019	0.013	0.006
	0.0511	11.617	4.011	0.011		
	0.0514	11.617	4.008	0.008		

Table S2. The results of the Rietveld analysis conducted for diffractograms from HT-XRD measurement of SmSr(Co,Fe,Ga,Mn,Ni)$O_{4+\delta}$ (cooling run).

Temperature [°C]	Space group	$a = b$ [Å]	c [Å]	V_0 [Å^3]	a_0 [Å]	GoF	Rwp
1000	*I4/mmm*	3.8473	12.6261	186.887	5.7173	2.81	2.85
900	*I4/mmm*	3.8421	12.5973	185.960	5.7079	2.87	2.90
800	*I4/mmm*	3.8367	12.5670	184.991	5.6979	2.88	2.91
700	*I4/mmm*	3.8313	12.5365	184.017	5.6879	2.68	2.82
600	*I4/mmm*	3.8264	12.5066	183.114	5.6786	2.44	2.69
500	*I4/mmm*	3.8212	12.4775	182.195	5.6691	2.47	2.71
400	*I4/mmm*	3.8165	12.4486	181.321	5.6600	2.50	2.72
300	*I4/mmm*	3.8120	12.4212	180.493	5.6514	2.88	2.92
200	*I4/mmm*	3.8076	12.3940	179.683	5.6429	3.91	3.41
100	*I4/mmm*	3.8039	12.3710	179.001	5.6358	2.24	2.60
25	*I4/mmm*	3.8012	12.3539	178.503	5.6305	2.03	2.48

Table S3. The results of the Rietveld analysis conducted for diffractograms from HT-XRD measurement of NdSr(Co,Fe,Ga,Mn,Ni)$O_{4+\delta}$ (cooling run).

Temperature [°C]	Space group	$a = b$ [Å]	c [Å]	V_0 [Å^3]	a_0 [Å]	GoF	Rwp
1000	*I*4/*mmm*	3.8590	12.7046	189.194	5.7408	4.65	3.51
900	*I*4/*mmm*	3.8537	12.6757	188.250	5.7312	4.68	3.53
800	*I*4/*mmm*	3.8485	12.6460	187.297	5.7215	4.77	3.56
700	*I*4/*mmm*	3.8433	12.6161	186.357	5.7119	4.47	3.44
600	*I*4/*mmm*	3.8381	12.5843	185.383	5.7020	4.35	3.41
500	*I*4/*mmm*	3.8332	12.5542	184.465	5.6925	4.03	3.28
400	*I*4/*mmm*	3.8284	12.5239	183.561	5.6832	3.97	3.25
300	*I*4/*mmm*	3.8238	12.4949	182.696	5.6743	3.96	3.27
200	*I*4/*mmm*	3.8196	12.4675	181.890	5.6659	3.82	3.20
100	*I*4/*mmm*	3.8154	12.4413	181.109	5.6578	4.98	3.68
25	*I*4/*mmm*	3.8125	12.4245	180.594	5.6524	4.85	3.67

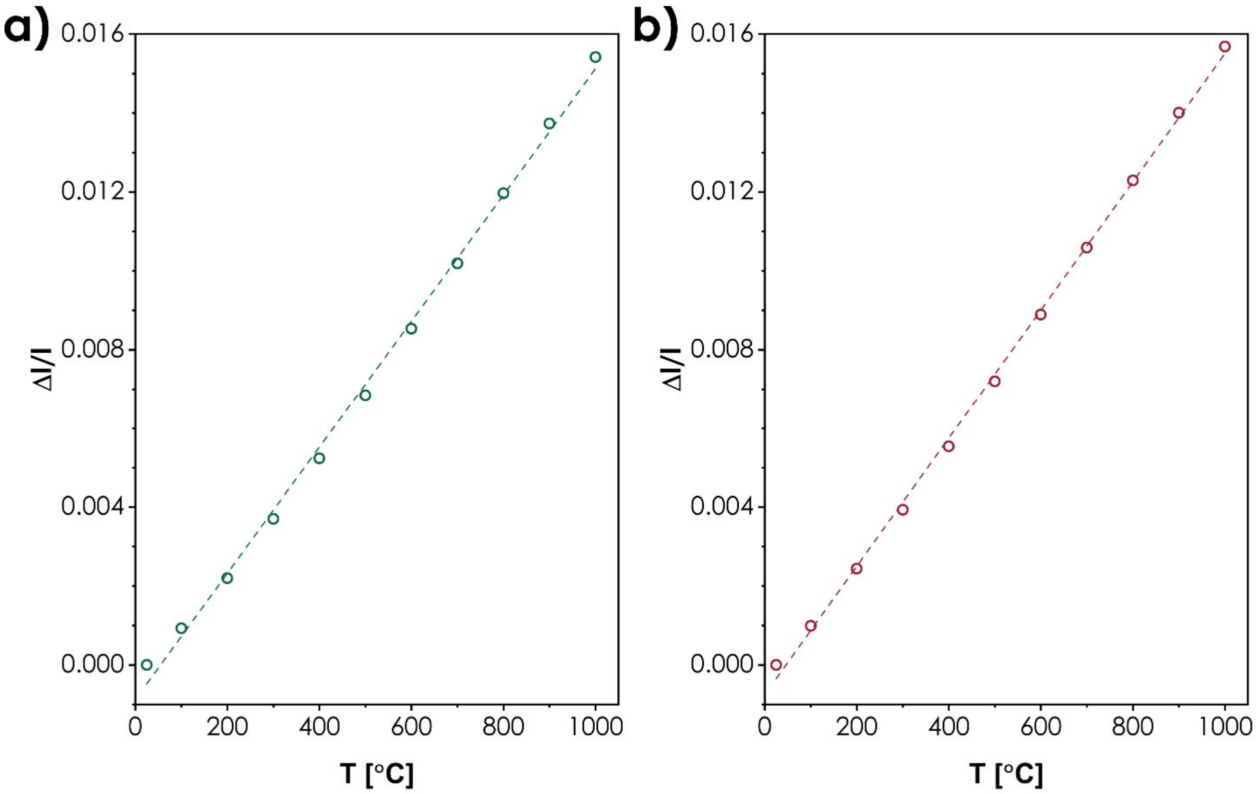

Figure S7. Thermal expansion behavior determined from the unit cell expansion during HT-XRD measurement for: (**a**) SmSr(Co,Fe,Ga,Mn,Ni)$O_{4+\delta}$; (**b**) NdSr(Co,Fe,Ga,Mn,Ni)$O_{4+\delta}$.
